# Climate-induced patterns of activity and nest conditions select for genes associated with growth and maturation in a widespread lizard species

**DOI:** 10.21203/rs.3.rs-9941634/v1

**Published:** 2026-06-12

**Authors:** Christopher Parkinson, Michael Sears, Matthew McTernan

**Affiliations:** Clemson University; Arizona State University; Clemson University

**Keywords:** Climate, Biophysics, Genomics, Selection, Life History

## Abstract

How species adapt to local climates across their ranges remains a central question in ecology and evolutionary biology. Though phenotypic responses to climate are well documented, the genetic basis for these adaptive responses remains largely unresolved. Here, we integrate biophysical and genomic methods to identify genomic variants associated with climate-induced patterns of activity and nest conditions across the range of the Eastern fence lizard (*Sceloporus undulatus*). We first use a biophysical model to estimate potential annual activity and incubation temperatures for nests across the species’ range. The biophysical parameters were then used in gene-environmental association models to identify candidate genes underlying adaptation to climate. The biological functions of candidate genes were assessed through enrichment analyses. Lastly, we use genome-wide linkage disequilibrium to identify evidence of co-selection or epistatic interactions on candidate genes. In general, activity time decreased with latitude, while the range of nest temperatures increased with latitude. However, mean nest temperatures do not correlate with latitude, longitude, or elevation. Genomic analyses identified 2040 candidate genes, of which 48 enriched for two KEGG pathways: the calcium signaling pathway and the gonadotropin-releasing hormone pathway. Calcium signaling dictates cell proliferation and migration through the effects of growth factors (e.g., growth and development), while gonadotropin-releasing hormone dictates the development of sexual traits and the timing of sexual maturity. Phenotypic evidence from previous studies indicates that selection on life history traits, such as body size and age at maturation, underlie adaptation to climate in ectotherms, and our results provide genetic support for this notion.

## INTRODUCTION:

Understanding how species adapt to local climates throughout their distribution remains a primary goal of ecological and evolutionary biology, particularly as the global climate continues to shift (Franks and Hoffmann, 2012; Stein et al., 2013; Sears et al., 2019). Species occupy a realized climatic niche, where their physiology has evolved to suit the environment and enable population persistence (Vandermeer, 1972). However, many species encounter a variety of climates, either due to expansion into new areas or changes in local climate conditions. Studies investigating the adaptive responses of animals to climate have documented putatively adaptive responses in phenology, life history, behavior, and physiology (Crozier and Hutchings, 2014; Urban et al., 2014). However, while phenotypic responses to climate have been well documented, the genetic basis of these adaptive responses remains largely unresolved (Urban et al., 2024). A better understanding of the genetics that facilitate adaptation to climate may prove critical, as many species globally are experiencing population declines due to rapid climate change (Urban, 2015, 2024) and may rely on evolutionary rescue to avoid extinction (Hoffmann and Sgró, 2011; Carlson et al., 2014). Further, genomic studies investigating climate adaptation can reveal insights into less easily observed phenotypic adaptations, providing a better understanding of how life adapts to climate and improving predictions and conservational applications of adaptive responses to climate change (Meek et al., 2022; Urban et al., 2024).

A key aspect of climate adaptation involves animals' ability to meet their living costs (Wild et al., 2025). These costs can be described as an energy budget that must be allocated to growth, maintenance, reproduction, activity, and storage (Sousa et al., 2010). Within a given environment, selection should dictate how energy is allocated across costs to maximize fitness (Stearns, 1992). To fund the cost of living, animals must navigate spatiotemporal heterogeneity in environmental temperatures to 1) avoid dangerous temperature extremes (Sunday et al., 2014), and 2) maintain sufficient activity such that food acquisition and processing rates can fund energetic demands (Congdon, 1989; Kearney, 2012). For ectotherms in particular, this relationship is paramount, as they rely directly on environmental heat sources to modulate body temperature (T_b_), and the capacity to be active and acquire food is temperature-dependent (Adolph and Porter, 1993; Sinervo et al., 2010). Consequently, species that span climate gradients often show concordant variation in life history traits: cooler climates that restrict activity produce slower-growing individuals that mature at larger body sizes, whereas warmer climates produce faster-growing individuals that mature at smaller body sizes (Morrison and Hero, 2003; Angilletta et al., 2004). Larger body sizes often increase fitness by increasing fecundity, while earlier maturation at smaller body sizes trades off fecundity for a higher probability of survival to first reproduction (Healy et al., 2019). If this “slow-fast continuum” of life history traits is adaptive, then activity time provides a direct estimate of a major selective pressure imposed by climate.

Selection by climate is further complicated in species with complex life cycles (Kingsolver et al., 2011; Levy et al., 2015). While activity results from mobile individuals selectively moving through their environment, early life stages that are immobile (such as embryos in eggs) passively experience environmental conditions. Developmental processes are temperature-dependent in ectotherms, with higher temperatures increasing the rate of development but reducing developmental efficiency, whereas cooler temperatures either slow or arrest development entirely (Andrews et al., 1997; Shine et al., 1997; Carlo et al., 2018). As a result, hotter incubation conditions result in earlier hatching times but smaller individuals. Conversely, cooler conditions result in delayed hatching but larger hatchlings. Mothers can choose nest conditions and thus select nest sites that optimize the development and survival of their offspring (Kolbe and Janzen, 2002; Warner and Andrews, 2002a). However, nest conditions can nonetheless vary across different climates despite maternal choice of nest site (Angilletta et al., 2009; Carlo et al., 2018). As a result, selection may adapt embryos across climates to optimize growth and development within their specific nest conditions. Further, the date of oviposition and length of incubation dictate the amount of potential activity hatchlings have to acquire energy and grow prior to the onset of winter conditions (Sniegula et al., 2024). Thus, incubation conditions and the knock-on effects on hatchling and adult phenotypes (Jonsson et al., 2022) likely pose a strong selection pressure that can be used to understand adaptation to climate in species with complex life cycles.

**Figure 1.**
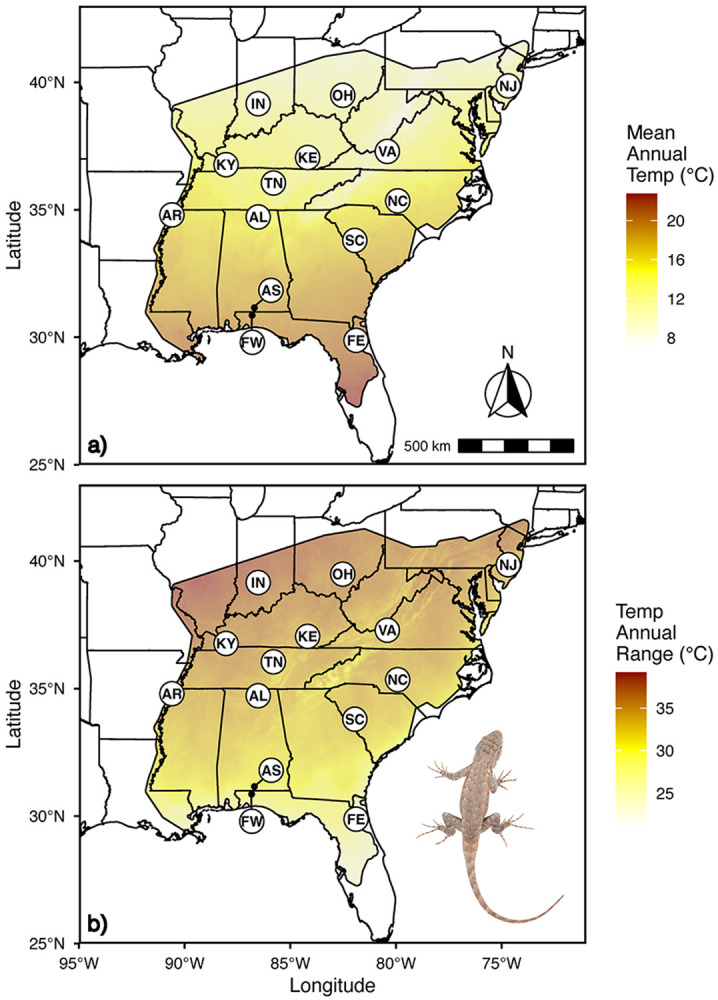
A) Map of the locations for the fourteen populations of *Sceloporus undulatus* used in this study. a) Mean annual temperatures and b) annual temperature range were plotted to illustrate climatic patterns across the species’ range. Names and locations of each site are shown as points on the map. The data used for these maps were obtained from the WorldClim2 (Fick and Hijmans, 2017) database for illustrative purposes. Photo credit for the *S. undulatus* specimen goes to Diego R. Quirola.

The Eastern fence lizard (*Sceloporus undulatus*) provides a model system for studying genetic adaptation to climate. The species inhabits a wide range of climates across the eastern United States (Fig. 1), and a wealth of research has identified phenotypic variation that putatively adapts populations to local climates. Adaptive responses in *S. undulatus* seem to center on life-history traits, with individuals in cooler climates growing more slowly, reaching sexual maturity later, and having larger body sizes than those in warmer climates (Tinkle and Ballinger, 1972; Angilletta et al., 2004; Kearney, 2012). Nest conditions vary across climates (Angilletta et al., 2009; Carlo et al., 2018), and survival rates are generally low (Tinkle and Ballinger, 1972; Andrews et al., 2000). Thus, there is potential for strong selection on developmental and growth processes during early life stages such that the emergent pattern of life histories is adaptive across climates. Additionally, life histories appear fixed across populations, indicating a genetic basis for these traits (Niewiarowski and Roosenburg, 1993; Niewiarowski, 1995). The recent publication of a *S. undulatus* reference genome (Westfall et al., 2021) now allows for a ‘genome up’ view through which we can identify adaptive traits and/or support previous studies aimed at understanding phenotypic adaptation to climate in this species.

In this study, we integrate biophysical and genomic methods to identify genetic variants and associated genes under selection in climates across the species distribution of the *S. undulatus*. We first use a biophysical model to estimate potential annual activity and nest conditions for populations across the full species’ range. We then use these estimates of activity and nest conditions as predictors to identify genetic variants putatively under selection across populations using two genome-environment association (GEA) models. Genes harboring adaptive variants are input into gene ontology and Kyoto Encyclopedia of Genes and Genomes (KEGG) enrichment analyses to identify biological functions that may underlie adaptation to climate. Lastly, we use genome-wide linkage disequilibrium to identify evidence of co-selection or epistatic interactions acting on candidate genes across the genome. Altogether, our study leverages an integrative approach to identify the genetic basis of adaptation to climate in a widespread ectothermic species.

## METHODS:

### Sample Collection and Variant Calling

We sampled 189 *S. undulatus* across 14 sites spanning the full range of climates experienced across the species distribution (Fig. 1)(Table S1). Of these, we sequenced 130 samples *de novo*, while we downloaded an additional 59 whole-genome sequences from the National Center for Biotechnology Information (accession code PRJNA656311)(Assis et al., 2025). We extracted genomic DNA from tail tips for wild-caught individuals and muscle from museum specimens using the DNeasy Blood and Tissue Kit (#69506, Qiagen, Inc.). We prepared whole-genome DNA libraries using the NEBNext Ultra II for DNA Library Prep kit (E6177L, NEBLabs, Inc.), with indexes provided by the NEBNext Oligos for Illumina kit (E7600S, NEBLabs, Inc.). We sequenced the 130 total *S. undulatus* genomic libraries on an Illumina NovaSeq 6000 S4 flow cell and an Illumina NextSeq P3 flow cell, with sequencing done to a target depth of 20x coverage.

We assessed read quality off the sequencers using FastQC v0.12.1 (Andrews, 2010). Both adapter sequences and low-quality reads (Phred score < 20) were trimmed using Trim Galore v0.6.10 (Krueger, 2015). We then aligned trimmed reads to the SceUnd_v1.1 reference genome for *S. undulatus* (Westfall et al., 2021) using BWA-MEM v0.7.17 (Li, 2013). We called variants from the aligned reads using the GATK best-practices pipeline (Van der Auwera et al., 2013), which reduces potential bias due to batch effects through joint variant calling. VCF files produced by the GATK pipeline (GATK v4.6.1.0, Picard v3.3.0) were then filtered using VCF_tools_ v0.1.17 (Danecek et al., 2011). We removed single nucleotide polymorphisms (SNPs) with minor allele frequency <5%, minimum sequencing depth <10x, maximum sequencing depth >50x (to account for alignment errors or PCR artifacts), and with missing data in more than 5% of individuals. We additionally pruned the dataset for physically linked SNPs using the software BCFtools v1.21with the +prune plugin (Danecek et al., 2021). Variants with an r^2^ > 0.50 were removed within a 10kb sliding window. The resulting filtered VCF file contained 1,409,447 high-quality SNPs for use in downstream analyses. We then annotated the VCF using SNPEff (Cingolani et al., 2012).

### Nest Condition Estimates:

To account for potential selection imposed by geographic variation in nest incubation temperatures across the species’ range, we estimated 20-year averages of nest conditions for two time periods: 1941-1960 and 2001-2020. Nest temperature was assumed to be equal to soil temperature, which we estimated using the *NicheMapR* R package (Kearney and Porter, 2017). We downloaded climate data from the ERA5 climate dataset (Hersbach et al., 2020) and calculated soil temperatures using the “micro_era5()” function in *NicheMapR*. We used temperatures at a soil depth of 6cm, which is the typical nest depth for *S. undulatus* (Warner and Andrews, 2002a; Angilletta et al., 2009; Carlo et al., 2018). To determine suitable nest conditions at each site, we first calculated nest temperatures across a range of shade conditions: 0%, 10%, 20%, 30%, 40%, 50%, 60%, 70%, 80%, and 100%. For each shade condition, we calculated the average temperature for each hour of the year across the 20 years for each period.

To determine potential oviposition dates, we assumed mothers do not oviposit until minimum nest temperatures are equal to or greater than 18°C, based on measurements of nest temperatures from the literature (Warner and Andrews, 2002a; Angilletta et al., 2009; Carlo et al., 2018), and knowledge that developmental rates slow down or cease at temperatures less than 18°C (Christian et al., 1986; Andrews et al., 1997). We then parameterized potential nest conditions using estimated developmental time and expected survival rates across shade conditions, based on nest temperatures. We used a previously published developmental time model to estimate rates of development (Levy et al., 2015):

$$E=\left(\frac{0.00081+0.00067\cdot t_{soil}}{24}\right)$$

where *E* is the hourly developmental rate, and *t_soil_* refers to the soil temperatures for a given shade value at 6 cm depth for each time point in the nest temperature data. If development was not completed before the nest conditions became too cold to continue, we categorized the nest as unsuitable. Further, we binned embryo survivorship based on data from the literature: nests that reached maximum temperatures of 42°C and above resulted in 0% embryonic survival, nests that reached maximum temperatures of 38-42°C resulted in 60% embryonic survival, and maximum temperatures below 38°C resulted in 80% survival (Levy et al., 2015; Carlo et al., 2018). Shade values that maximized embryonic survival and allowed for complete development of embryos were considered optimal nests. We averaged optimal nest temperatures across shade conditions to obtain a single representative annual optimal nest temperature for each site within each time period. From these optimal nests, we calculated mean nest temperatures in the 1900s and 2000s, the change in mean nest temperatures between the 1900s and 2000s, the range of nest temperatures in the 1900s and 2000s, and the change in range of nest temperatures from the 1900s to 2000s. Further, we used degree-day calculations to estimate changes in the total heat received by embryos in suitable nests between 1941-1960 and 2001-2020 (Zalom et al., 1983). Degree days (°*D*) for 2000s nests relative to 1900s nests were calculated relative to mean and maximum nest temperatures using the following equation:

$$D=\frac{\left(\sum_{day=1,hour=0}^{day=i,hour=j}{\left(t_{h}-t_{0}\right)}_{i,j}\right)}{24}$$

where *D* = sum of °*D*, *t_h_* = mean or maximum nest temperatures (°C) at time *i* on day *j* for nests in the 2000s, and *t_0_* = mean or maximum nest temperatures at time *i* on day *j* for nests in the 1900s.

### Activity Time Estimates:

We modeled annual activity time for both adult lizards and hatchlings at each site using standard biophysical models applied to site-specific climate data (Campbell and Norman, 1998; Sears et al., 2011). We used *NicheMapR* to calculate surface soil temperatures, 2-cm air temperatures, and 2-m air temperatures from the ERA5 climate data downloaded for 1941-1960 and 2001-2020. We calculated air and soil temperatures in full sun and shade to capture the maximum and minimum temperature profiles at each site. From these temperatures, we extracted monthly maximum and minimum temperatures and averaged across the two 20-year time periods for input into our biophysical model. We calculated radiation using standard equations (Gates, 1980).

We estimated annual activity time using the approach outlined in Sears et al. (2011). We calculated annual activity as the duration an individual could remain active within its preferred T_b_ range on a representative day of each month, and used spline interpolation to estimate activity for all days of the year. We calculated body temperature for each representative day by solving the following equation every minute over 24 hours:

$$T_{b2}=T_{e}+e^{\left(\frac{-t}{\tau }\right)}(T_{b1}-T_{e})$$

where *T_e_* is the operative temperature of the individual, *t* is the elapsed time in minutes, *τ* is the time constant of the individual, *T_b1_* is the initial body temperature, and *T_b2_* is the final body temperature. We calculated operative temperatures as follows:

$$T_{e}=T_{a}+\frac{R_{abs}-\epsilon_{s}\sigma {\left(T_{a}+273.15\right)}^{4}}{c_{p}(g_{r}+g_{Ha})}$$

where *R_abs_* is the total radiation absorbed, *e_s_* is the surface emissivity, *σ* is the Stefan-Boltzmann constant, *c_p_* is the specific heat of air at constant pressure, *g_r_* is the radiative conductance, and *g_Ha_* is the boundary layer conductance for heat. We used 2 cm air temperature (i.e., the approximate mid-body height of lizards off the substrate) for *T_a_*. We calculated longwave radiation from the ground and sky using ground surface temperatures and 2 m air temperatures, respectively. We modeled wind at 0.1 m/s.

For adult activity, we parameterized the model for a representative 10 g Eastern fence lizard with a snout-vent length (SVL) of 65 mm and venter height of 1 cm above the substrate (Tinkle and Ballinger, 1972). We modeled hatchlings with a body mass of 0.5 g, SVL of 25 mm, and a venter height of 0.5 cm (Tinkle and Ballinger, 1972; Warner and Andrews, 2002b; Carlo et al., 2018). After 30 days of activity, we assumed that hatchlings had grown and we modeled them as having a body mass of 2.5 g, an SVL of 41 mm, and a venter height of 0.75 cm (Tinkle and Ballinger, 1972; Warner and Andrews, 2002b). We assumed lizards at each site to be active at a given time if the calculated range of possible T_b_ values encompassed part of the T_b_ range of active lizards (29-35°C)(Andrews, 1998). We estimated adult activity for the entire year, whereas hatchling activity began only upon completion of development, as determined by the developmental time model for the nest conditions at each site. We summed potential activity time to calculate the average potential annual activity for each site over the two 20-year periods. As *S. undulatus* is heliothermic, we limited potential activity to only times when the sun was above the horizon (e.g., cosine of the zenith angle > 1). We estimated potential adult and hatchling activity in the 1900s and 2000s, and changes in potential adult and hatchling activity from 1900s to 2000s.

Further, we calculated time above CT_max_ (TACT_max_) as a metric of potential heat stress at each site. Using *T_e_* estimates for an individual in full sun, we summed the minutes for which *T_e_* > CT_max_ for this species. We set CT_max_ at 42°C (Crowley, 1985; Claunch et al., 2021) and estimated TACT_max_ for adults and hatchlings in the 1900s and 2000s, and the change in TACT_max_ for adults and hatchlings between 1900s and 2000s.

### Assessment of Biophysical Variables

We first looked for patterns of activity and nest variables across the species’ range using geographic predictors. We used linear mixed models to test whether latitude, longitude, or elevation predicted patterns or changes over time in activity or nest conditions. Interactive effects for latitude, longitude, and elevation were included in the models. We then assessed the degree of covariation among biophysical predictor variables using the *corrplot* R package (Wei and Simko, 2024). Due to the high degree of covariation between the activity and nest variables (Fig. S1), we performed a principal component analysis (PCA) to generate PC axes for use in our genomic analyses. The PCA was centered and scaled to account for unit differences among variables. Further, we included latitude, longitude, and elevation in the PCA to account for geographic variation among populations. We determined the number of PC axes to include as predictors for our genomic analyses by assessing a scree plot (Fig. S2). We then visualized variable loadings on the PC axes using the *ggbiplot* R package (Vu and Friendly, 2024).

### Gene-Environmental Association Modeling

We used two gene-environmental association (GEA) approaches to detect directional selection on SNPs in response to the biophysical predictors: BayeScEnv (Villemereuil and Gaggiotti, 2015) and latent-factor mixed models (LFMM2)(Caye et al., 2019). We chose these models due to their ability to account for deviations from neutrality caused by confounding variables, specifically population structure. Following the approach implemented by (Salloum et al., 2022), we combined results across models using the geometric mean of q-values to identify candidate SNPs. Significance was assessed based on a mean q-value less than 0.05. Genes that harbored candidate SNPs within 5kb were considered candidate genes putatively adapting populations to differences in activity and nest conditions across the species’ range.

BayeSceEnv is an F_ST_-based genome scan program that leverages genetic and environmental differences among populations to identify genetic variants putatively under selection. The program uses a Markov Chain Monte Carlo (MCMC) algorithm to compare three competing models: a neutral model, and two locus-specific models. The neutral model acts as the null against which the locus-specific models are tested. The two locus-specific models then account for deviations from neutrality due to: 1) divergent selection driven by the predictor variable, or 2) other process different from local adaptation, such as allele surfing or background selection. We ran the BayeScEnv program with the following parameters: 10 pilot runs with 2,000 iterations each; a thinning interval of 10; 5,000 output iterations; and a burn-in length of 10,000. We used default priors for the MCMC chain. We used the *coda* package in R to ensure the chain reached convergence with low autocorrelation (Plummer et al., 2006). Convergence was tested using the Heidelberg and Welch’s convergence diagnostic procedure, and autocorrelation was tested using the “autocorr.diag” function.

The LFMM2 model uses a latent variable regression to model allele frequencies as a function of the environmental variable of interest across populations, while accounting for a latent factor—in this case, population structure. All analyses were implemented using the *LEA* package in R (Frichot and François, 2015). We first assessed population structure in our data using sparse non-negative matrix factorization, implemented by the “snmf” function. We ran the “snmf” function for K values 1 through 20, with 10 repetitions each. Because the LFMM2 model does not tolerate missing data, we used the resulting snmfProject to impute missing values in our genotype matrix with the “impute” function. The imputed SNP dataset was then regressed over the biophysical predictors using the “lfmm2” function, and significant SNPs were identified using the “lfmm2_test” function. Resulting p-values were converted to q-values to correct for multiple comparisons using the R package *qvalue* (Storey et al., 2023).

### Gene Ontology and KEGG Enrichment Analyses

We used gene ontology (GO) and Kyoto Encyclopedia of Genes and Genomes (KEGG) enrichment to determine whether biological processes or pathways are enriched among candidate genes identified in the GEA analysis. We input the full list of candidate genes into *ShinyGO* (Xijin Ge et al., 2020), an online resource for GO and KEGG enrichment analyses and graphing. We used the *S. undulatus* genome as the background reference. A false discovery rate (FDR) threshold of 0.05 was used to assess the significance of assigned GO terms and KEGG pathways.

### Genome-wide Linkage Disequilibrium

Selection on complex phenotypic traits or traits under pleiotropic control may result in covariance in allele frequencies across the genome. Therefore, we tested for patterns of interchromosomal linkage disequilibrium (LD) among candidate SNPs identified by the GEA analyses. Since linkage analyses increase exponentially with each additional SNP, we downsampled to one SNP per candidate gene for this analysis. We used the R package *GWLD* (Zhang et al., 2023) to calculate LD among all pairs of SNPs, using the standard r^2^ metric. SNPs found in the top 99.5th percentile in the resulting r^2^ distribution were designated as in significant disequilibrium. Further, we compared the r^2^ distribution generated from the candidate SNPs with 100 randomly generated SNP datasets to assess whether rates of LD in the candidate SNPs differed from background levels. We randomly sampled SNPs from the full SNP dataset using BCFtools within a custom bash script, and then entered them into the *GWLD* pipeline. The resulting r^2^ distributions were compared with the candidate SNP distribution using the Wilcoxon rank-sum test and the Fisher-Pitman permutation test, implemented in base R and the R package *coin* (Hothorn et al., 2008), respectively.

## RESULTS:

### Estimates of activity time and nest conditions

**Figure 2.**
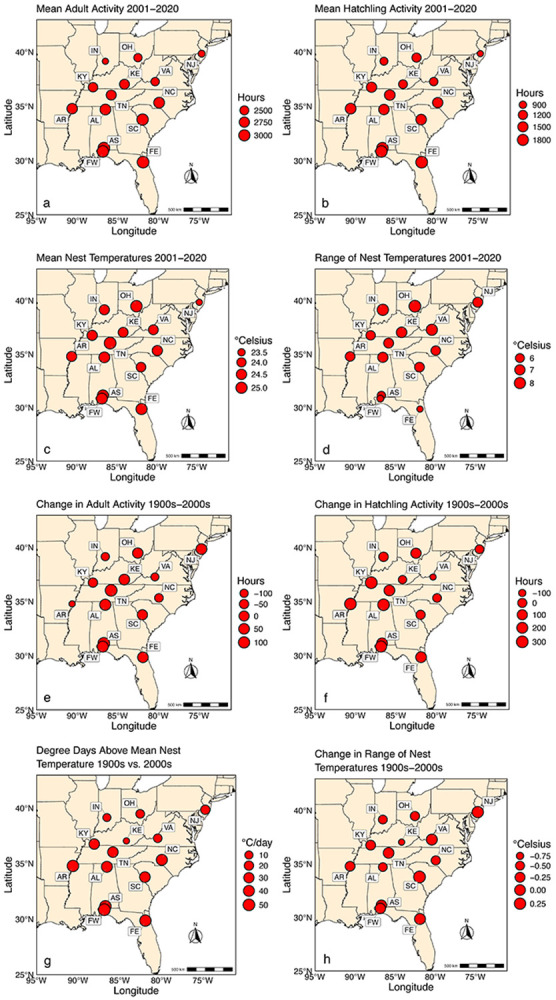
Multiple biophysical variables describing activity patterns and nest conditions were estimated to use as predictors for gene-environmental association analyses. The maps show patterns of these variables across populations, with some exemplars shown here: a) patterns of annual adult activity averaged from 2001-2020, b) patterns of annual hatchling activity averaged from 2001-2020, c) mean nest temperatures averaged from 2001-2020, d) range of nest temperatures averaged from 2001-2020, e) change in patterns of adult activity from 1941-1960 to 2001-2020, f) change in patterns of hatchling activity from 1941-1960 to 2001-2020, g) degree days (C°/day) above mean nest temperature from 1941-1960 to 2001-2020, and h) change in range of nest temperatures from 1941-1960 to 2001-2020.

Latitude strongly predicted activity time estimates across both adult and hatchling life stages (Table 1). Within each time and life stage, our model predicted that populations at lower latitudes had greater potential annual activity than those at higher latitudes (Fig. 2a-b). For adults, our model estimated activity times ranging from ~2280 hrs for the lowest potential activity at the Indiana site to ~3170 hrs of potential activity at the eastern Florida site. However, an interaction between longitude and elevation predicted a change in potential activity for adults between the 1900s and 2000s (Table 1), with the largest increase in potential activity between centuries occurring in the Tennessee site (149 hrs, a 5.8% increase from 1900s to 2000s) (Fig. 2e). Likewise, an interaction between latitude, longitude, and elevation predicted a change in potential activity. Our model predicted that hatchlings at mid-latitude sites west of the Appalachian Mountains would experience the largest increase in potential activity time, with increases of up to 313 hrs (a 25% increase) in western Kentucky (Fig. 2f).

Time above CT_max_ (TACT_max_) also varied with latitude for both adult and hatchling life stages and across both time periods. Populations at lower latitudes had higher potential TACT_max_ than those at higher latitudes, with TACT_max_ ranging from 1286-2091 hrs in adults and from 209-1107 hrs in hatchlings. Change in TACT_max_ also varied with latitude for adults, with sites at low latitudes showing an increase in potential TACT_max_ from the last century to this century, the largest increase being 281 hrs in western Florida. However, for hatchlings, an interaction between latitude, longitude, and elevation predicted the change in TACT_max_. In general, TACT_max_ increased more at lower latitudes and elevations than at higher ones, with Arkansas showing the largest increase (223 hrs).

The relationship between nest conditions and geographic predictors was more complicated than with aspects of activity or TACT_max_. Latitude and elevation predicted degree days above mean nest temperatures between the 1900s and 2000s. While all nests were predicted to experience warming (*i.e.*, more degree days)(Fig. 2g), the degree of warming was predicted to be higher at lower latitudes and lower elevations than at higher latitudes or elevations (e.g., ~52 degree days in western Florida versus 9.3 degree days in Indiana). Concordantly, a change in mean nest temperatures between centuries was predicted by interactions between both latitude and longitude with elevation. Generally, sites at lower latitudes or lower elevations were predicted to have warmed more than those at higher latitudes or elevations (e.g., a 0.34°C increase in western Florida versus a 0.11°C increase in Indiana). The range of nest temperatures also varied predictably with latitude within both centuries, with higher ranges of nest temperatures experienced at higher latitudes (e.g., 8.2°C range in Indiana versus 5.1°C range in western Florida) (Fig. 2d). However, the change in the range of nest temperatures between centuries was not predicted by any geographic predictor (Fig. 2h). Mean nest temperatures (Fig. 2c) and degree days above maximum temperatures also did not vary with any geographic predictor variable.

All biophysical model outputs are available in supplemental materials as a table (Table S2). Maps of variables not shown in Fig. 2 are also available (Fig. S3).

### PCA of activity time and nest conditions

**Figure 3.**
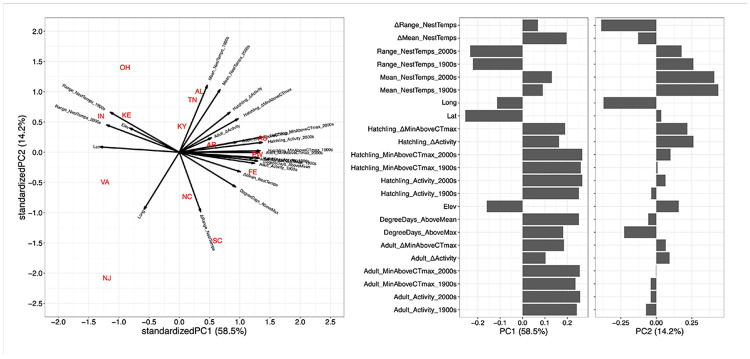
A principal component analysis (PCA) was used to reduce multidimensionality among the correlated biophysical predictor variables. The locations of each site in PC spaces are shown in the biplot on the left. The loadings for each variable onto the PC axes are shown by the bar plots on the right. Variables that correlate with latitude mostly loaded onto PC1, while multiple variables associated with nest conditions and hatchling activity loaded onto PC2.

There was a high degree of correlation between latitude, longitude, elevation, and our estimated variables for activity, TACT_max_, and nest conditions (Fig. S1). As such, we conducted a PCA to reduce collinearity in our predictor variables. After assessing the scree plot (Fig. S2), we selected the first two PC axes as predictors for genome-environmental association models. The first PC axis (PC1) accounted for 58.5% of the variance and primarily loaded latitude, elevation, and variables associated with activity time, TACT_max_, nest temperature range, and change in mean nest temperature (Fig. 3). PC2 accounted for 14.2% of the variation and primarily loaded longitude and variables associated with nests and hatchling activity, including mean nest temperatures, change in mean nest temperatures, nest temperature range, change in range nest temperatures, change in hatchling activity, change in TACT_max_, and degree days above maximum nest temperatures.

### Genome-Environmental Association Analyses

**Figure 4.**
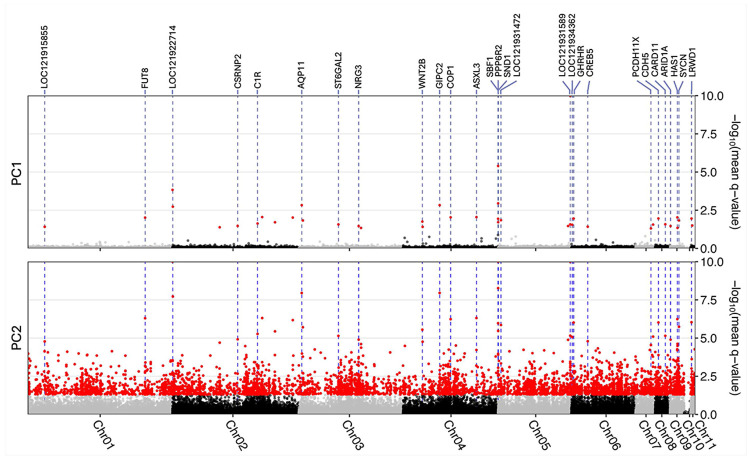
The output of the GEA analyses identified 41 candidate SNPs associated with PC1 and 4,974 candidate SNPs associated with PC2. There were 27 candidate genes identified across both PC1 and PC2, and their locations and names are shown. Q-values were calculated using the geometric mean of the outputs from both LFMM2 and BayeScEnv, and a significance threshold of 0.05 was employed to identify candidate SNPs significantly associated with PC1 or PC2 (candidates shown in red).

The number of candidate SNPs varied considerably between PC1 and PC2. Using the geometric mean of the q-values from the BayeScEnv and LFMM2 models, we identified 41 candidate SNPs associated with PC1 and 4,974 candidate SNPs associated with PC2 (Fig. 4). However, there was overlap between SNPs identified by PC1 and PC2, as 40 of the 41 candidate SNPs associated with PC1 were also identified by PC2. Of the 41 candidate SNPs identified by PC1, 28 were associated with genes, hereafter referred to as “candidate genes”. Likewise, of the 4,974 candidate SNPs identified by PC2, 3,341 were associated with 2039 candidate genes. These candidate gene sets represent 0.13% and 9.3% of the 21,891 genes found on the chromosomes of the SceUnd_v1.1 *S. undulatus* reference genome, respectively. The overlap in candidate SNPs between PC1 and PC2 led to a concordant overlap in candidate genes, with 27 of the 28 candidate genes identified by PC1 also identified by PC2 (Fig. 4). Genes identified by both PC axes were dispersed relatively evenly across the genome, indicating the potential for polygenic selection across multiple loci (Fig. S4), though relatively few were found on chromosome 10, the putative X chromosome for this species. Due to the high overlap between PC1 and PC2 candidate genes, these datasets were combined for enrichment and genome-wide linkage analyses, yielding 2040 candidate genes for the GO enrichment and genome-wide linkage analysis.

The analyses of the MCMC chain produced by the BayeScEnv model showed both convergence (Cramer-von-Mises statistic, all p>0.05) and low autocorrelation (all R < 0.05). For the LFMM2 model, the sNMF analysis identified K=10 as the most supported K value (Fig. S5). The run with the lowest cross-entropy value for K=10 from the snmfProject was used to impute missing data before input into the LFMM2 model.

### Gene Ontology and KEGG Enrichment Results

Using the 2040 candidate genes identified across PC1and PC2, we identified 48 genes that significantly enriched for two KEGG pathways: Gonadotropin-Releasing Hormone Signaling Pathway (GnRH pathway, Path:sund04912), and Calcium Signaling Pathway (Ca^2+^ pathway, Path:sund04020)(Table 2). For the GnRH pathway, 22 of the 106 pathway genes were identified in our candidate gene dataset, whereas for the Ca^2+^ pathway, 39 out of 260 genes were identified. Of the 48 candidate genes, 13 were associated with both KEGG pathways, whereas 9 were associated with the GnRH pathway and 26 were associated with only the Ca^2+^ pathway. These genes include five calcium voltage-gated channel subunits (CACN), four mitogen-activated protein kinases (MAPK), three calcium/calmodulin-dependent protein kinases (CAMK), and three phospholipase C proteins (PLC). No significantly enriched GO terms were found in our analysis.

### Genome-Wide Linkage Analysis

**Figure 5.**
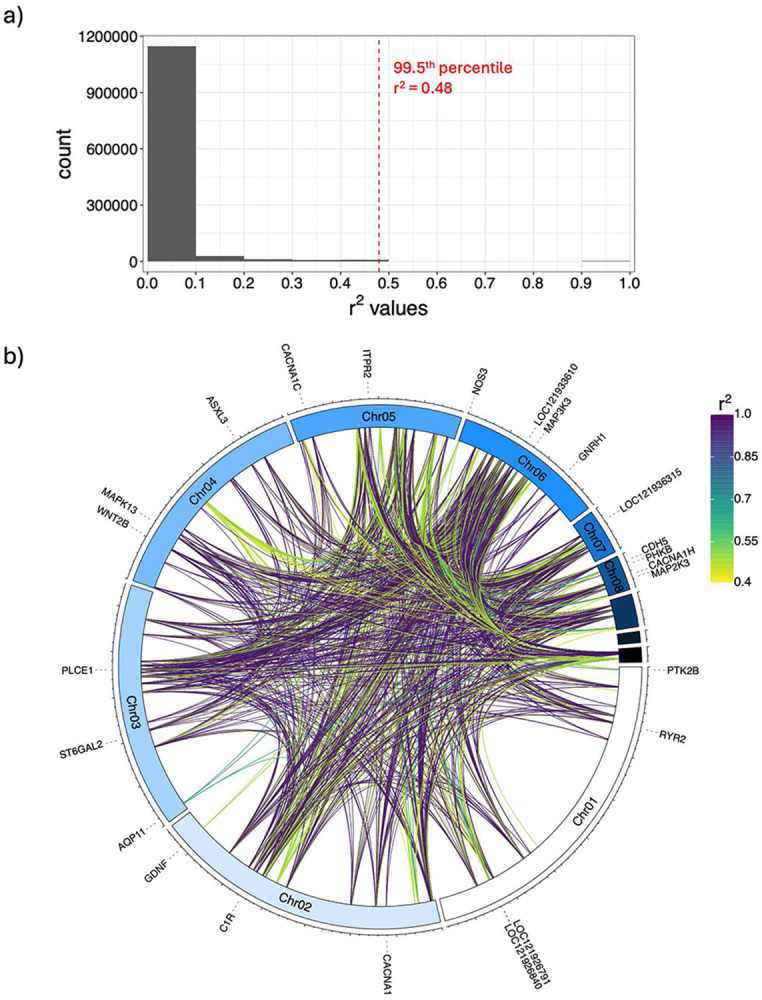
We used genome-wide linkage disequilibrium to identify correlations between candidate genes identified using LFMM2 and BayeScEnv. A) Histogram of the distribution of linkage values (r^2^) for the 1,206,681comparisons between the 2040 candidate SNPs. A large majority of the SNPs showed no evidence of linkage disequilibrium (mean r^2^ = ~0.017). B) Candidate genes that harbor SNPs in significant linkage (r^2^ > 0.48, the 99.5^th^ percentile) are plotted with linkage values shown by the color-coded connections. The locations of 18 genes that significantly enrich for calcium signaling and gonadotropin-releasing hormone pathways are shown (LOC121933610 “ADCY1-like”, LOC121936315 “CACNA1B-like”, CACNA1C, CACNA1D, CACNA1H, GDNF, GNRH1, ITPR2, MAP2K3, MAP3K3, MAPK13, NOS3, PHKB, LOC121926791 “PLA2G4D-like”, LOC121926840 “PLA2G4E-like”, PLCE1, PTK2B, and RYR2). Additionally, the location of six candidate genes identified across both PC1 and PC2 are plotted (CDH5, CR1, AQP11, ST6GAL2, WNT2B, ASXL3).

One SNP per candidate gene was used to assess interchromosomal linkage between candidate genes (see Methods). Of the 1,206,681 linkage calculations across the 2040 candidate SNPs used for this analysis, an overwhelming majority were found to not be in linkage disequilibrium (r^2^ < 0.10)(Fig. 5A). The resulting r^2^ distribution was heavily right-skewed, with a mean of ~0.017 and a 99^th^ percentile of 0.44. These values were consistently greater than those obtained from linkage analyses using randomly selected SNPs (Table S3). On average, the random SNP datasets had a mean r^2^ of ~0.008 and a 99^th^ percentile of 0.17. Fisher-Pitman Permutation and Wilcoxon rank sum tests supported the notion that the candidate SNPs were in higher linkage than random, with 100/100 permutation tests and 97/100 Wilcox tests showing significant differences between the candidate SNP r^2^ distribution and the r^2^ distribution generated from random SNPs.

High interchromosomal linkage among candidate genes can indicate co-selection in response to environmental selection or selection on epistatic interactions among genes (Zhang, 2017). To identify candidate genes with high linkage, we used a linkage threshold at the 99.5^th^ percentile, corresponding to an r^2^ threshold of 0.48. Above this threshold, we found a total of 639 SNPs in linkage disequilibrium with at least one other candidate gene, constituting 31.3% of the 2040 genes identified by our GEA analyses. Of the 639 candidate genes found to be in high linkage, 18 were previously identified to significantly enrich for KEGG pathways, while 6 were common genes identified across both PC1 and PC2 predictors in the GEA analyses. These represent 37.5% of the genes identified as enriching for KEGG pathways and 22.2% of the common genes identified by both PC1 and PC2 in the GEA analyses, respectively. In total there were 111 genes in significant linkage with either one of the KEGG enriching genes or one of the genes common to both PC1 and PC2 (Fig. 5b). Of the KEGG-enriching genes, 11 were involved in linkage groups with four or more candidate genes (Table S4): CACNA1C, CACNA1D, CACNA1H, LOC121936315 (CACNA1B-like protein), GNRH1, ITPR2, LOC121933610 (ADCY1-like protein), MAP3K3, MAPK13, NOS3, and PLCE1. Of these, the highest number of significant linkages to other candidate genes were found in CACNA1C (n=10), CACNA1H (n=10), MAP3K3 (n=13), MAPK13 (n=33), and PLCE1 (n=30). Further, MAPK13 and PLCE1 were in significant linkage with each other, and were linked to the same 29 candidate genes. A table of all the KEGG enriching genes and the genes to which they are linked is available in Supplemental Materials (Table S4).

## DISCUSSION:

Selection imposed by climate induces local adaptation, but the genetics underlying these adaptations remains poorly understood. Knowledge of the adaptive traits and underlying genetics may prove critical to conservation efforts, as many species will likely rely on adaptive evolution to persist in rapidly changing climates (Hoffmann and Sgró, 2011; Urban et al., 2024). Here, we used biophysical estimates of activity time and nest conditions across the Eastern fence lizard (*Sceloporus undulatus*) species’ range to identify genomic signatures of selection. Activity time dictates rates of energy intake and expenditure and thus represents a fundamental control over the ability of organisms to fund the cost of living (Congdon, 1989; Wild et al., 2025), whereas nest conditions affect rates of growth and development, with downstream consequences to survival rates and life histories (Andrews et al., 2000; Carlo et al., 2018; Jonsson et al., 2022). Our biophysical approaches identified a general pattern that potential activity time decreases with latitude, while the range of nest temperatures increases with latitude, and mean nest temperatures do not correlate with latitude, longitude, or elevation. When input into GEA analyses, the suite of biophysical variables identified a large number of candidate genes, with 48 that enriched for two KEGG pathways: the calcium signaling pathway and the gonadotropin-releasing hormone pathway. Calcium signaling is associated with cell proliferation and migration through the effects of growth factors (e.g., growth and development)(Berridge, 1995; Whitaker, 2006), while gonadotropin-releasing hormone dictates the development of sexual traits and the timing of sexual maturity (Licht, 1979). Phenotypic evidence from previous studies suggests that selection on life history traits such as body size and age at maturation underlies adaptation to climate in ectotherms; thus, our results provide genetic support for this notion (Crozier and Hutchings, 2014; Urban et al., 2014).

Life history theory focuses on the tradeoffs in resources allocated to survival, growth, and reproduction (Stearns, 1992). When resources are limited, selection should act to optimize the allocation of resources across these costs, thereby maximizing fitness within a given environment (Stearns, 1992). Optimization of resource allocation appears to have led to the co-evolution of life history traits, producing axes of variation in survival to maturity, size at maturity, timing of reproduction, and reproductive investment (Healy et al., 2019). The results of our study suggest that selection on growth processes and age at maturation underlie adaptation to climates across the range of *S. undulatus*. Specifically, we identified 48 genes significantly enriched in two KEGG pathways: the calcium signaling pathway and the gonadotropin-releasing hormone (GnRH) pathway. An important role of calcium signaling is to regulate cell proliferation and migration (Berridge, 1995). This may be particularly important during embryogenesis and early development, as calcium signaling has been implicated in regulating cell proliferation, migration, axis formation, and organogenesis in animal embryos (Whitaker, 2006). Selection on aspects of the calcium signaling pathway may therefore represent adaptations to nest conditions across the range of *S. undulatus*. Our biophysical models identified variation in both mean and range of nest temperatures experienced across sites, with changes in nest temperatures occurring over the last 60-80 years. As nest temperatures directly affect incubation time and size at hatching (Andrews et al., 1997; Shine et al., 1997; Carlo et al., 2018), selection may be acting on processes that control growth and development pathways to optimize these processes within the range of temperatures the embryo experiences. Indeed, a previous common garden study using populations of *S. undulatus* from across the species’ range identified differences among populations in incubation time and embryonic growth efficiency (Oufiero and Agilletta, Jr., 2006). The knock-on effects of these early developmental processes may, at least in part, affect post-hatching growth rates and adult body size patterns observed across the species’ range (Jonsson et al., 2022).

Age at maturation post-hatching appears to be another adaptive life history trait in *S. undulatus*. Warmer climates at lower latitudes facilitate more activity time, which in turn increases opportunities for food acquisition and reproductive output (Adolph and Porter, 1993). However, greater activity comes with a trade-off: more active populations in southern latitudes experience lower survival rates (likely due to greater exposure to predators)(Adolph and Porter, 1993; Angilletta et al., 2004). Survival to maturation has been directly linked to age at maturation, such that populations with lower survival rates mature earlier (Adolph and Porter, 1996), increasing the likelihood of reproducing before death (Stearns and Crandall, 1981). As such, populations of *S. undulatus* in warmer climates have evolved rapid post-hatching growth rates and earlier maturation at a smaller body size relative to cooler-climate populations (Tinkle and Ballinger, 1972; Adolph and Porter, 1996). Our study identified genes in the GnRH pathway as under selection across our study populations, providing genomic evidence supporting the view that age at maturation is an adaptive trait selected by climate. The GnRH pathway regulates aspects of maturation in animals by stimulating gonadotropins, specifically follicle-stimulating hormone and luteinizing hormone (Counis et al., 2005). These hormones then affect sexual traits, including gonadal maturation, gamete formation, and the production of sex-specific hormones such as testosterone and progesterone (Licht, 1979). Therefore, selection on genes within the GnRH pathway may act to control the timing of maturation across populations of *S. undulatus*. Further, signal transmission of GnRH acts through calcium signaling (Catt and Stojilković, 1989), linking the GnRH pathway to the calcium-signaling pathway genes identified in our study. Indeed, many of the genes we identified act in both the calcium signaling and GnRH pathways (Table 2). Therefore, while we postulate that calcium signaling genes control adaptive responses underlying embryonic growth and development under different nest conditions, it is possible that the signatures of selection we detect in calcium-signaling genes may also act in conjunction with the GnRH pathway to control age at maturation.

Some gene families identified in this study have previously been implicated in controlling aspects of growth, development, and sexual maturation. The mitogen-activated protein kinase (MAPK) gene family mediates intercellular signal transduction (Seger and Krebs, 1995). Activation of a single kinase by an upstream mitogen or growth factor triggers a cascade of downstream kinases that ultimately alter gene expression, inducing multiple responses, including cell proliferation, differentiation, migration, and cell death (Krens et al., 2006). The MAPK signaling pathway has been identified as controlling multiple aspects of vertebrate development (Krens et al., 2006; Craig et al., 2008), as well as maturation of oocytes in animals (Meinecke and Krischek, 2003; Ni et al., 2015), due to the interaction of MAPK activity and maturation-promoting factor (MPF)(Kotani and Yamashita, 2002). Calcium/calmodulin-dependent protein kinases (CAMK) has also been implicated in controlling aspects of organismal growth (Braun and Schulman, 1995; Kahl and Means, 2003). This family of kinases functions as a critical component of the calcium signaling pathway, acting as effectors that translate calcium influx into changes in cellular function through interactions with a wide range of substrates (Braun and Schulman, 1995). The action of calcium/calmodulin-dependent kinases has been directly implicated in controlling aspects of muscle growth (Al-Shanti and Stewart, 2009) and neurological development (Wayman et al., 2008). Further, CAMK genes have been implicated in control aspects of oocyte maturation in yaks (Yang et al., 2024) and pigs (Xu et al., 2009), providing a means by which they can potentially influence the age of maturation. Lastly, phospholipase C (PLC) genes link the effects of extracellular messengers, such as growth factors and hormones, to intracellular activities by generating inositol 1,4,5-trisphosphate and diacylglycerol as secondary messengers (Nakamura and Fukami, 2017). The phenotypic effects of PLC activity putatively include effects on body size (Zhang et al., 2024) and embryonic development (Zhou and Hildebrandt, 2009). Due to the fundamental roles the above gene families play as components of the calcium signaling and GnRH pathways, as well as their influence on aspects of embryonic development, organismal growth, and gamete maturation, these genes are strong candidates for underpinning the adaptive phenotypic responses of life histories to climate in *S. undulatus*.

The evolution of whole-organism phenotypes, such as those described by life-history traits, can involve complex interactions among a large number of genes (Whiting et al., 2022). To that end, selection can act on epistatic interactions between genes within networks or pathways, resulting in patterns of interchromosomal linkage (Zhang, 2017). We investigated this possibility and found elevated linkage in candidate genes, exceeding background levels across the genome. Among the candidate genes exhibiting elevated linkage were multiple MAPK genes and the PLCE1 gene, which were previously discussed as candidates underlying patterns of life-history variation across populations of *S. undulatus*. In particular, MAPK13 (found on chromosome four) and PLCE1 (found on chromosome three) were found to form a linkage group with 29 other candidate genes, including those critical for embryonic development (HOXB1, STAT3, DMXL1, HDAC9, MED12)(Levy and Lee, 2002; McGraw et al., 2003; Shin et al., 2008; Hubert and Wellik, 2023; Vontell et al., 2025). Among these genes, a well-known interaction has been described between KMT and HOX genes, in which KMT (lysine N-methyltransferases) directly regulates HOX gene expression during embryonic development (Milne et al., 2002). While it may be difficult to definitively link the effects of mutations across all candidate genes, the overall evidence points to processes that control patterns of embryonic development. Thus, it seems likely that selection via nest conditions on processes of growth and development may underlie adaptive patterns of growth and development as life histories become optimized across climates.

Our study investigated the genetics that underlie adaptation to climate in the *S. undulatus,* a well-studied model species in ecology and evolution. Life history varies predictably across climate in *S. undulatus* (Tinkle and Ballinger, 1972; Adolph and Porter, 1993), and other studies have identified various life history traits as putatively under selection by climate in other ectothermic species (Crozier and Hutchings, 2014; Urban et al., 2014). By integrating biophysical and genomic approaches, we identified a suite of genes putatively under selection by climate-induced patterns in activity and nest incubation conditions. Many of the candidate genes identified in this study have known effects on processes of growth and development in vertebrates. In particular, we identified candidate genes associated with the calcium signaling and gonadotropin-releasing hormone pathways, which have known effects on cell proliferation, embryonic development, and sexual maturation. Our results ultimately suggest that selection acting on early life stages (e.g., from embryonic development to sexual maturity) drives patterns of life history in response to climate in a wide-spread lizard species.

## Supplementary Material

This is a list of supplementary files associated with this preprint. Click to download.

- SupplementaryMaterials.zip
- Table1PredictorsLatLongElevLinearModelsResultsTable.xlsx
- Table2KEGGEnrichmentGenes.xlsx
